# Relationships among Height, Weight, Body Mass Index, and Age in Taiwanese Children with Different Types of Mucopolysaccharidoses

**DOI:** 10.3390/diagnostics9040148

**Published:** 2019-10-14

**Authors:** Hsiang-Yu Lin, Chung-Lin Lee, Pao Chin Chiu, Dau-Ming Niu, Fuu-Jen Tsai, Wuh-Liang Hwu, Shio Jean Lin, Ju-Li Lin, Tung-Ming Chang, Chih-Kuang Chuang, Shuan-Pei Lin

**Affiliations:** 1 Department of Medicine, MacKay Medical College, New Taipei City 252, Taiwan; lxc46199@ms37.hinet.net; 2 Department of Pediatrics, MacKay Memorial Hospital, Taipei 100, Taiwan; 3 Department of Medical Research, MacKay Memorial Hospital, Taipei 100, Taiwan; 4 MacKay Junior College of Medicine, Nursing and Management, Taipei 100, Taiwan; 5 Department of Medical Research, China Medical University Hospital, China Medical University, Taichung 400, Taiwan; 6 Institute of Biomedical Sciences, MacKay Medical College, New Taipei City 252, Taiwan; 7 Department of Pediatrics, MacKay Memorial Hospital, Hsinchu 300, Taiwan; clampcage@yahoo.com.tw; 8 Institute of Clinical Medicine, National Yang-Ming University, Taipei 100, Taiwan; dmniu1111@yahoo.com.tw; 9 Department of Pediatrics, Kaohsiung Veterans General Hospital, Kaohsiung 800, Taiwan; pcchiu@vghks.gov.tw; 10 Department of Pediatrics, Taipei Veterans General Hospital, Taipei 100, Taiwan; 11 Department of Pediatrics, China Medical University Hospital, Taichung 400, Taiwan; d0704@mail.cmuh.org.tw; 12 Department of Pediatrics, National Taiwan University Hospital, Taipei 100, Taiwan; hwuwlntu@ntu.edu.tw; 13 Department of Pediatrics, Chi Mei Medical Center, Tainan 700, Taiwan; shiojean@gmail.com; 14 Department of Pediatrics, Chang Gung Memorial Hospital at Linkou, Taoyuan 33305, Taiwan; jllin001@gmail.com; 15 Department of Pediatric Neurology, Changhua Christian Children’s Hospital, Changhua 500, Taiwan; 128658@cch.org.tw; 16 Department of Biological Science and Technology, College of Biological Science and Technology, National Chiao Tung University, Hsinchu 300, Taiwan; 17 College of Medicine, Fu-Jen Catholic University, Taipei 100, Taiwan; 18 Department of Infant and Child Care, National Taipei University of Nursing and Health Sciences, Taipei 100, Taiwan

**Keywords:** body mass index, growth, height, mucopolysaccharidosis, weight

## Abstract

Background: Children with mucopolysaccharidosis (MPS) generally appear unaffected at birth but may develop multiple clinical manifestations including profound growth impairment as they grow older. Each type of MPS has a variable age at onset and variable rate of progression, however, information regarding growth in Asian children is limited. Methods: This retrospective analysis included 129 Taiwanese patients with MPS (age range, 0.7 to 19.5 years, median age, 7.9 years) from eight medical centers in Taiwan from January 1996 through December 2018. Results: The mean *z* scores for the first recorded values of height, weight, and body mass index in the patients’ medical records were −4.25, −1.04, and 0.41 for MPS I (*n* = 9), −2.31, 0.19, and 0.84 for MPS II (*n* = 49), −0.42, 0.08, and −0.12 for MPS III (*n* = 27), −6.02, −2.04, and 0.12 for MPS IVA (*n* = 30), and −4.46, −1.52, and 0.19 for MPS VI (*n* = 14), respectively. MPS IVA had the lowest mean *z* scores for both height and weight among all types of MPS, followed by MPS VI, MPS I, MPS II, and MPS III, which showed the mildest growth retardation. Both *z* scores for height and weight were negatively correlated with increasing age for all types of MPS (*p* < 0.01). Of 32 patients younger than 5 years of age, 16 (50%), and 23 (72%) had positive *z* scores of height and weight, respectively. A substantial number of younger patients with MPS I, II, III, and IVA had a positive height *z* score. The median age at diagnosis was 3.9 years (*n* = 115). Conclusions: The patients with MPS IVA had the most significant growth retardation among all types of MPS, followed by MPS VI, MPS I, MPS II, and MPS III. The height and weight of the MPS patients younger than 2–5 years of age were higher than those of healthy individuals, however, their growth significantly decelerated in subsequent years. Understanding the growth curve and potential involved in each type of MPS may allow for early diagnosis and timely management of the disease, which may improve the quality of life.

## 1. Introduction

Mucopolysaccharidoses (MPSs) are a group of lysosomal storage disorders resulting from deficiencies in specific enzymes which catalyze the stepwise degradation of glycosaminoglycans (GAGs). Eleven known enzymes are related to the catabolism of GAGs, including dermatan sulfate (DS), heparan sulfate (HS), keratan sulfate (KS), chondroitin sulfate (CS), and hyaluronic acid. The accumulation of GAGs in cells causes progressive severe tissue and organ dysfunction. The clinical signs and symptoms in these patients include short stature, coarse facial features, vision and hearing impairment, airway obstruction, recurrent respiratory infections, cardiovascular disease, hepatosplenomegaly, umbilical, and inguinal hernias, communicating hydrocephalus, spinal cord compression, developmental delay, and skeletal deformities (dysostosis multiplex). Patients with the severe forms of MPS I and II also have somatic and cognitive involvement. MPS III is characterized by cognitive and neurological impairment as well as mild somatic involvement. MPS IVA manifests as short stature, skeletal dysplasia, odontoid hypoplasia, joint hypermobility, and ligamentous laxity without cognitive impairment. MPS VI presents with purely somatic manifestations similar to those observed in MPS I and II, with no cognitive involvement [1,2,3,4].

The mechanism of growth impairment and short stature in the different types of MPS may be due to defects in the growth plate, including GAG deposition in bone and cartilage, impaired osteoblast function, the disorganized structure of the growth plate, hypertrophic chondrocytes, and decreased matrix deposition. Compared to the growth retardation of the skeletal system, other organs may still grow normally, which leads to an imbalance in growth manifestations with a prominent forehead, tracheal obstruction, spinal cord compression, hepatosplenomegaly, and dwarfism with short neck and short trunk [5]. Children with MPS generally appear unaffected at birth but may develop multiple clinical manifestations including profound growth impairment as they grow older. Each type of MPS has a variable age at onset and variable rate of progression. Growth measurement is critical in the evaluation of therapeutic efficacy and disease progression in MPS.

The main treatments for MPS disorders include enzyme replacement therapy (ERT) and hematopoietic stem cell transplantation [6]. Although these treatments cannot cure the diseases, they can improve or alleviate the progression of the natural course. Knowledge about the natural history and growth pattern of MPS is important when assessing the therapeutic efficacy. Previous studies have described the growth pattern in MPS patients, however, these reports only focused on a specific type of MPS without comparing the results with other types of MPS in a single population [5,7,8,9,10,11,12,13,14,15,16,17,18,19,20,21,22]. The purpose of this study was to compare growth patterns among Taiwanese patients with different types of MPS. The first recorded data on height, weight, body mass index (BMI), and age, and birth weight, as well as the diagnostic age, were analyzed.

## 2. Materials and Methods

### 2.1. Ethics Approval and Consent to Participate

All procedures followed were in accordance with the ethical standards of the responsible committee on human experimentation (institutional and national) and with the Declaration of Helsinki of 1975, as revised in 2000. The Institutional Review Board of MacKay Memorial Hospital approved this study (the approved date: 30 March 2015; the project identification code: 14MMHIS281). Written informed consent was obtained from a parent for the children less than 18 years of age and from the patients themselves if they were 18 years or older.

### 2.2. Study Population

In this retrospective study, we enrolled 129 Taiwanese patients with MPS (age range, 0.7 to 19.5 years, nine with MPS I, 49 with MPS II, 27 with MPS III, 30 with MPS IVA, and 14 with MPS VI, 89 males and 40 females, mean age, 9.1 ± 5.3 years, median age, 7.9 years) from January 1996 through December 2018 from eight medical centers in Taiwan, including MacKay Memorial Hospital, Kaohsiung Veterans General Hospital, Taipei Veterans General Hospital, China Medical University Hospital, National Taiwan University Hospital, Chi Mei Medical Center, Changhua Christian Children’s Hospital, and Chang Gung Children’s Hospital. No cases of MPS VII were ascertained during the study period. The diagnosis of the type of MPS was confirmed by specific enzyme activity assay in serum, leukocytes and/or skin fibroblasts, two-dimensional electrophoresis of urinary GAGs, and/or identification of a pathogenic mutation [23]. MPS I patients were classified into three syndromes: Hurler syndrome (severe), Hurler-Scheie syndrome (intermediate), and Scheie syndrome (attenuated). For patients with MPS II, the severe form was defined according to the presence of cognitive impairment compared with the mild form (without cognitive impairment). All 44 patients with MPS IVA or VI in this study had typical forms with clinical features observed before five years of age. None of the patients had received ERT or hematopoietic stem cell transplantation before entering this study. The first recorded data on height, weight, BMI, and age, and birth weight, as well as the diagnostic age, were analyzed. Standard deviation scores (*z* scores) for height, weight, and BMI were calculated using standard growth tables for the Taiwanese population [24]. A *z* score was derived by subtracting the population mean from each individual’s raw score and then dividing the difference by the standard deviation of the population. Cubic regression was used to fit the curves of this cohort.

### 2.3. Statistical Analysis

Descriptive statistics were performed, and the results are presented as mean ± standard deviation unless otherwise indicated. The relationships among age and height, weight, and BMI in the patients with different types of MPS were determined using Pearson’s correlation coefficients (*r*), and significance was tested using Fisher’s *r-z* transformations. All statistical analyses were performed using SPSS version 11.5 (SPSS Inc., Chicago, IL, USA), and differences with *p* < 0.05 were considered to be statistically significant.

## 3. Results

**Figure 1:**
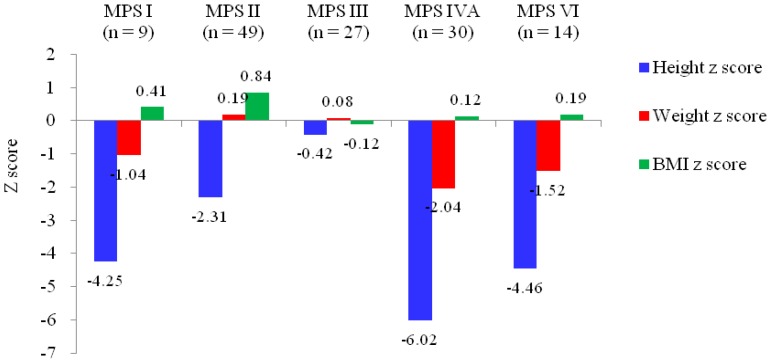
The mean *z* scores of height, weight, and body mass index (BMI) of 129 Taiwanese children with different types of mucopolysaccharidosis (MPS).

**Figure 2:**
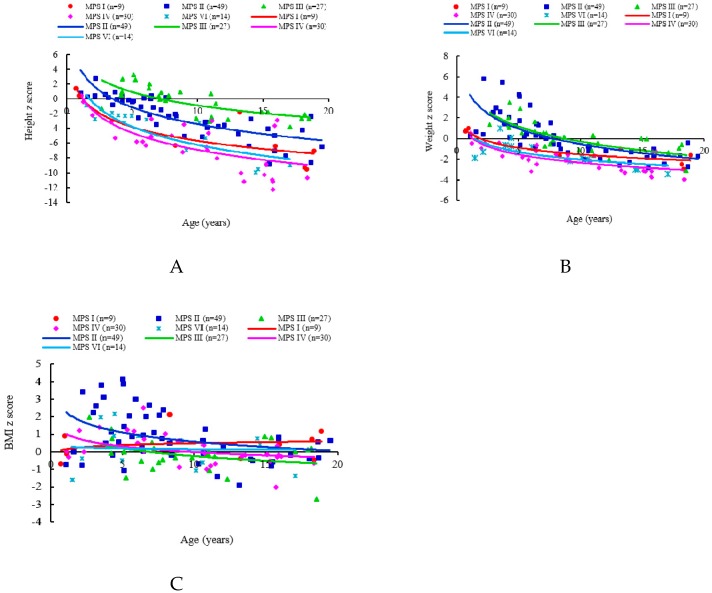
Associations of age and *z* scores of height (**A**), weight (**B**), and body mass index (BMI) (**C**) of 129 Taiwanese children with different types of mucopolysaccharidosis (MPS). The lines are derived from the cubic regression on this cohort.

**Figure 3:**
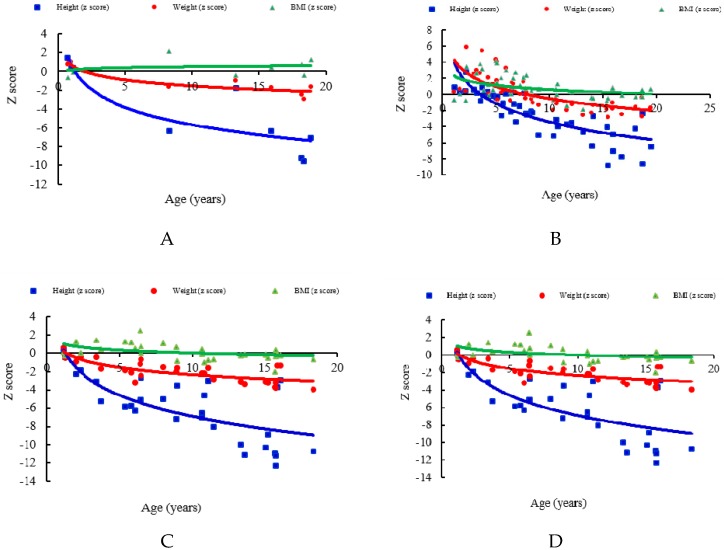

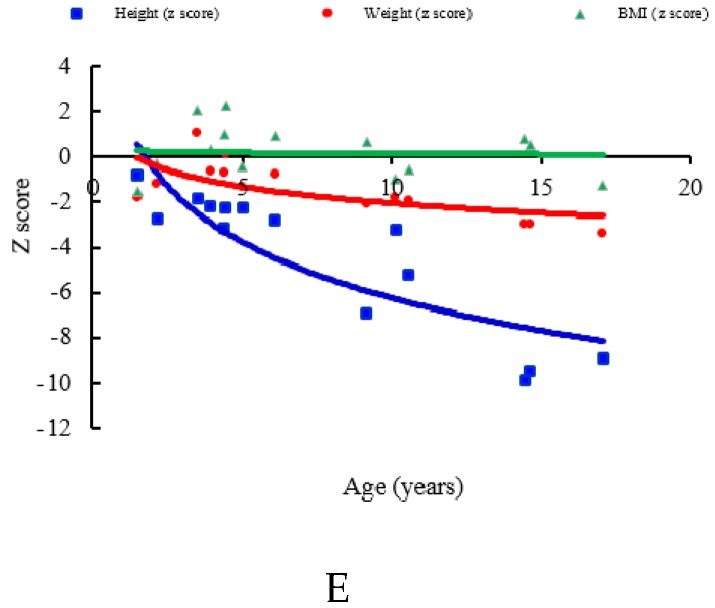
Associations of age and *z* scores of height, weight, and body mass index (BMI) of 129 Taiwanese children with different types of mucopolysaccharidosis (MPS). (**A**) MPS I (*n* = 9), mean age: 10.6 ± 8.0 years, (**B**) MPS II (*n* = 49), mean age: 8.7 ± 5.1 years, (**C**) MPS III (*n* = 27), mean age: 9.5 ± 4.8 years, (**D**) MPS IVA (*n* = 30), mean age: 9.5 ± 5.2 years, (**E**) MPS VI (*n* = 14), mean age: 7.6 ± 5.0 years. The lines are derived from the cubic regression on this cohort.

**Figure 4:**
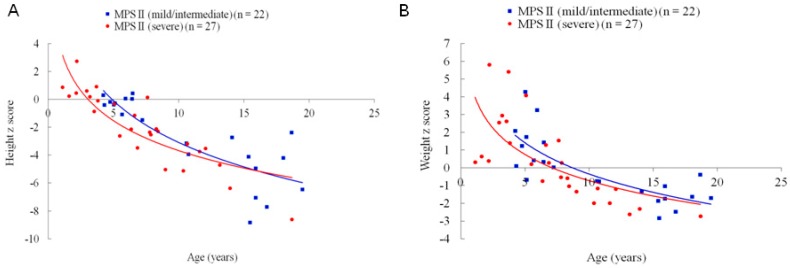
Associations of age and *z* scores of height (**A**) and weight (**B**) of children with MPS II mild/intermediate form (*n* = 22) and MPS II severe form (*n* = 27). The lines are derived from the cubic regression on this cohort.

The mean *z* scores for the first recorded values of height, weight and BMI in the patients’ medical records were −4.25, −1.04, and 0.41 for MPS I (*n* = 9, including one with Hurler, six with Hurler-Scheie, and two with Scheie syndrome), −2.31, 0.19, and 0.84 for MPS II (*n* = 49, including 27 with the severe form and 22 with the mild/intermediate form), −0.42, 0.08, and −0.12 for MPS III (*n* = 27), −6.02, −2.04, and 0.12 for MPS IVA (*n* = 30), and −4.46, −1.52, and 0.19 for MPS VI (*n* = 14), respectively. Both *z* scores for height and weight were negatively correlated with increasing age for all types of MPS (*p* < 0.01). The BMI *z* scores of the patients with MPS II and MPS IVA were negatively correlated with increasing age (*p* < 0.01). The patients with MPS IVA had the lowest mean *z* scores for both height and weight among all types of MPS, followed by MPS VI, MPS I, MPS II, and MPS III, which showed the mildest growth retardation. The patients with MPS II had the largest mean *z* scores of weight (0.19) and BMI (0.84) among all types of MPS. The values of BMI in patients with all types of MPS were close to those of the normal population (Table 1 and Figure 1 and Figure 2). The growth trends of each type of MPS with the *z* scores for height, weight, and BMI are depicted in Figure 3. Table 2 and Figure 4 show the correlations among height, weight, and age of the 49 children with the mild/intermediate form (*n* = 22) or severe form (*n* = 27) of MPS II. The mean height *z* scores were −2.67 and −2.01 and the mean weight *z* scores were −0.11 and 0.42 for the mild/intermediate and severe form of MPS II, respectively. The mean age of 29 patients with a positive height *z* score was 4.6 years compared to 10.4 years in 100 patients with a negative height *z* score. Of 32 patients younger than five years of age, 16 (50%) and 23 (72%) had positive *z* scores of height and weight, respectively. All patients with MPS I, 67% with MPS II, 80% with MPS III, and 17% with MPS IVA had positive height *z* scores. However, none of six patients with MPS VI had a positive height *z* score. All patients with MPS I, II, and III, 17% with MPS IVA, and 33% with MPS VI had positive weight *z* scores (Table 3). Of the 65 patients with data on the birth weight, the mean birth weight was 3429 g for males (*n* = 49) and 3178 g for females (*n* = 16). The mean birth weights of 65 patients with different types of MPS are shown in Table 4. Of the 115 patients with data on the age at diagnosis, the median age at a confirmed diagnosis of all types of MPS was 3.9 years. The patients with MPS I (Hurler syndrome) had the youngest median age at diagnosis of 0.7 years, followed by MPS I (Hurler-Scheie syndrome) (2.2 years), MPS II severe form (3.0 years), MPS VI (3.2 years), MPS II mild/intermediate form and MPS IVA (4.2 years), MPS III (4.5 years), and MPS I (Scheie syndrome) had the oldest median age at diagnosis of 13.6 years (Table 5).

## 4. Discussion

To the best of our knowledge, this is the first report to describe the relationships among height, weight, BMI, and age of patients with various types of MPS for a single population. In our cohort, the patients with MPS IVA had the lowest mean *z* scores for both height and weight among all types of MPS, followed by MPS VI, MPS I, MPS II, and MPS III, which showed the mildest growth retardation. Both *z* scores for height and weight were negatively correlated with increasing age for all types of MPS, demonstrating the progressive nature of this disorder. The patients with MPS II had the largest mean *z* scores of weight and BMI among all types of MPS. Previous studies have reported the growth patterns of MPS using data collected from the global disease registry or from a single population, however, they only analyzed a specific type of MPS [7,8,9,10,11,12,13,14,15,16,17,18,19,20,21,22]. MPS I, II, III, IVA and VI have many similar clinical features, but also some specific manifestations. The clinical manifestations of the different types of MPS can be categorized into three groups depending on the type of GAG accumulation. The “visceral” group is caused by DS and includes MPS I, II, VI, and VII, with manifestations of coarse facial features, adenotonsillar hypertrophy, vision and hearing loss, upper airway obstruction, cardiac disease, hepatosplenomegaly, short stature, joint stiffness, and skeletal deformities. The “neurodegenerative” group is caused by HS and includes MPS III, MPS I (Hurler syndrome), and the severe form of MPS II, with presentations of cognitive decline, mental retardation, and behavioral disturbances. The “skeletal” group is caused by KS and includes MPS IVA, with manifestations of joint laxity, odontoid hypoplasia, genu valgum, extreme short stature, and skeletal dysplasia. The amounts of the affected GAG-derived disaccharides have been associated with the clinical presentations, onset, and severity of MPS disorders [25,26,27,28]. Settembre et al. [29] reported that GAG accumulation in lysosomes disrupts autophagy, which is important for chondrocyte metabolism during endochondral ossification, leading to chondrocyte viability and thus the development of skeletal abnormalities. We found that the MPS patients younger than 2–5 years of age were taller and heavier than the general population, however, their growth significantly decelerated as they grew older.

### 4.1. MPS I

A Polish study on the growth pattern in children with MPS I (*n* = 16) reported that until 2 years of age, the average height *z* scores for Hurler patients were above Polish reference charts. However, height velocities decreased in these children when they were older than 2 years of age compared to controls. The mean birth weight was 3430 g [7]. In our cohort, the mean *z* scores for the first recorded values of height, weight and BMI in their medical records were −4.25, −1.04, and 0.41 for nine patients with MPS I. However, all three MPS I patients younger than 5 years of age had positive height *z* scores. There was a trend of a decrease in growth velocity after 2–4 years of age (Figure 3A), and the mean birth weight was 3520 g (*n* = 5). Our results are consistent with those of the Polish study. D’Aco et al. [9] reported that the median age at diagnosis was 0.8 years for Hurler syndrome, 3.8 years for Hurler-Scheie syndrome, and 9.4 years for Scheie syndrome from MPS I Registry (*n* = 891). Similarly, in our study, the median age at diagnosis was 0.7 years for Hurler syndrome (*n* = 1), 2.2 years for Hurler-Scheie syndrome (*n* = 6), and 13.6 years for Scheie syndrome (*n* = 2).

### 4.2. MPS II

Previous studies have reported that children with MPS II tend to be taller and heavier than unaffected individuals for the first 3–5 years of age, and then their growth slows in subsequent years, reaching lower values in comparison with reference charts. No statistical differences in height and weight have been reported between patients with the attenuated and severe forms at any age [5,10,11,12]. Our results are in agreement with these findings (Table 2, Figure 3B and Figure 4). Patel et al. [11] reported a mean birth weight of 3350 g for 111 Japanese male patients with MPS II. Consistently, the mean birth weight was 3429 g for 34 male patients with MPS II in our cohort. Parini et al. [12] reported a median age at the diagnosis of MPS II of 3.0 years (*n* = 316) for those with the severe form, and 3.8 years for those with the attenuated form (*n* = 320). Similarly, in our study, the median age at the diagnosis of MPS II was 3.0 years for the severe form (*n* = 25), and 4.2 years for the mild/intermediate form (*n* = 22).

### 4.3. MPS III

Muschol et al. [16] reported that their cohort of German MPS III patients (*n* = 182) were taller than healthy children before 2–4 years of age, and that subsequently, their growth velocity decelerated after 4.5–5 years of age. Moreover, they were shorter than the reference group at 17.5 years of age. Our results are similar to theirs (Figure 3C). De Ruijter et al. [17] reported a mean birth weight of 3500 g for 118 MPS III patients in the Netherlands. In our cohort, the mean birth weight was 3104 g for 13 MPS III patients. Truxal et al. [18] reported a mean age of the diagnosis of MPS III of 3.4 years (*n* = 25). Consistently, the mean age at the diagnosis of MPS III in our patients was 4.6 years (*n* = 27).

### 4.4. MPS IVA

Short stature is a more significant presentation in MPS IVA than in other types of MPS [19]. Montaño et al. [20] reported that the mean birth weights for both male (3560 g) and female (3500 g) patients were similar to those of healthy individuals. The growth pattern is characterized by impaired growth velocity after one year of age, and growth usually stops by the age of 7–8 years [5,20]. In our study, the mean birth weight for the patients with MPS IVA was 3697 g (*n* = 7). During earlier childhood, growth in patients with MPS IVA is already diminished in comparison with other MPS types, as shown in our results (Figure 3D). Montaño et al. [30] reported a mean age at diagnosis of 4.7 years for patients in the International Morquio A Registry data (*n* = 311), which is similar to our results (4.8 years, *n* = 20).

### 4.5. MPS VI

Quartel et al. [22] reported that MPS VI patients had short stature and growth failure. In their study, patients were separated into two groups, those with slowly progressive and rapidly progressive disease, depending on urinary GAG levels of > or ≤200 μg/mg creatinine. After 4–5 years of age, growth of the rapidly progressive group slowed significantly compared with the slowly progressive group. The median height for those aged 18 years in the slowly progressive group was 144.1 cm compared with 109.3 cm in the rapidly progressive group. In our cohort, the urinary GAG levels of all 14 MPS VI patients were >200 μg/mg creatinine, which could be defined as rapid progression. The mean height *z* score was −4.46 (*n* = 14). Our results are consistent with Quartel et al.’s (Figure 3E). In addition, the following data for MPS VI patients was lacking in the literature. In our cohort, the mean birth weight of the patients with MPS VI was 3075 g (*n* = 6). None of the six patients with MPS VI who were younger than 5 years of age had a positive height *z* score. The median age at diagnosis was 3.2 years (*n* = 12).

### 4.6. Limitations

As a retrospective and cross-sectional study, not all clinical data were available for all of our subjects. The data of height and weight were recorded from the first medical record in the hospital. The small sample size of each type of MPS reflects the rare nature of this genetic disorder. Meanwhile, the degree of disease severity was quite wide, as was the age range. Further studies with larger cohorts and a longer follow-up period are warranted.

## 5. Conclusions

Growth retardation is a common feature in all types of MPS. MPS IVA showed the most significant growth retardation among all types of MPS, followed by MPS VI, MPS I, MPS II, and MPS III. Surprisingly, the growth pattern for a substantial portion of the children with MPS tended to be taller and heavier than unaffected individuals for the first 2–5 years of age, followed by a significant deceleration in subsequent years. There did not seem to be any significant association between central nervous system involvement and growth impairment in MPS II. Understanding the growth curve and potential involved in each type of MPS may allow for early diagnosis and timely management of the disease, as well as accurate assessments of the therapeutic efficacy, which may improve the quality of life. These findings and follow-up data can be used to develop the quality of care strategies for patients with MPS.

## Figures and Tables

**Table 1:** Associations of *z* scores of height, weight, and body mass index (BMI) and age of 129 Taiwanese children with mucopolysaccharidosis (MPS).

MPS Type	N	Age Range (Years)	Age (Years)	Height *z* Score	Height *z* Score vs. Age		Weight *z* Score	Weight *z* Score vs. Age		BMI *z* Score	BMI *z* Score vs. Age	
					*r*	*p*		*r*	*p*		*r*	*p*
MPS I	9	0.7–18.9	10.6 (8.0)	−4.25 (4.35)	−0.893	**0.001**	−1.04 (1.43)	−0.916	**0.0005**	0.41 (0.92)	0.120	0.758
MPS II	49	1.1–19.5	8.7 (5.1)	−2.31 (2.71)	−0.858	**<0.0001**	0.19 (2.10)	−0.728	**<0.0001**	0.84 (1.46)	−0.435	**0.002**
MPS III	27	2.7–18.5	9.5 (4.8)	−0.42 (1.98)	−0.751	**<0.0001**	0.08 (1.48)	−0.728	**<0.0001**	−0.12 (0.93)	−0.292	0.139
MPS IVA	30	1.1–18.4	9.5 (5.2)	−6.02 (3.46)	−0.736	**<0.0001**	−2.04 (1.15)	−0.749	**<0.0001**	0.12 (0.89)	−0.534	**0.002**
MPS VI	14	1.5–17.1	7.6 (5.0)	−4.46 (3.10)	−0.924	**<0.0001**	−1.52 (1.23)	−0.785	**0.0009**	0.19 (1.15)	−0.206	0.480

Data are mean (standard deviation). *p* < 0.05 are printed in bold.

**Table 2:** Associations of *z* scores of height and weight and age of 49 Taiwanese children with the mild/intermediate or severe forms of MPS II.

MPS II Subtype	N	Age (Years)	Height *z* Score	Height *z* Score vs. Age		Weight *z* Score	Weight *z* Score vs. Age		BMI *z* Score	BMI *z* Score vs. Age	
				*r*	*p*		*r*	*p*		*r*	*p*
MPS II (mild/intermediate)	22	10.5 (5.6)	−2.67 (2.87)	−0.839	**<0.0001**	−0.11 (1.83)	−0.763	**<0.0001**	0.66 (1.19)	−0.379	0.082
MPS II (severe)	27	7.2 (4.3)	−2.01 (2.58)	−0.915	**<0.0001**	0.42 (2.30)	−0.746	**<0.0001**	0.99 (1.65)	−0.488	**0.009**

Data are mean (standard deviation). MPS, mucopolysaccharidosis. *p* < 0.05 are printed in bold.

**Table 3:** The number of positive *z* scores for height and weight of 32 Taiwanese children younger than 5 years of age with different types of MPS.

MPS Type	N	n (Height *z* Score >0)	% (Height *z* Score >0)	n (Weight *z* Score >0)	% (Weight *z* Score >0)	n (BMI *z* Score >0)	% (BMI *z* Score >0)
I	3	3	100%	3	100%	1	33%
II	12	8	67%	12	100%	9	75%
III	5	4	80%	5	100%	4	80%
IVA	6	1	17%	1	17%	5	83%
VI	6	0	0%	2	33%	4	67%
Total	32	16	50%	23	72%	23	72%

MPS, mucopolysaccharidosis.

**Table 4:** The mean birth weight of 65 Taiwanese children with different types of mucopolysaccharidosis (MPS).

MPS Type	N	Mean Birth Weight (g)
MPS I	5	3520
MPS II	34	3429
MPS III	13	3104
MPS IVA	7	3697
MPS VI	6	3075
All	65	3365

**Table 5:** The median and mean ages at diagnosis of 115 Taiwanese children with different types of mucopolysaccharidosis (MPS).

MPS Type	N	Median Age at Diagnosis (Years)	Mean Age at Diagnosis (Years)
MPS I (H)	1	0.7	0.7
MPS I (H/S)	6	2.2	3.3
MPS I (S)	2	13.6	13.6
MPS II (severe)	25	3.0	3.3
MPS II (mild/intermediate)	22	4.2	4.8
MPS III	27	4.5	4.6
MPS IVA	20	4.2	4.8
MPS VI	12	3.2	3.4
Total	115	3.9	4.3

H, Hurler syndrome, H/S, Hurler-Scheie syndrome, S, Scheie syndrome.

## Abbreviations

MPS	mucopolysaccharidosis
GAGs	glycosaminoglycans
DS	dermatan sulfate
HS	heparan sulfate
KS	keratan sulfate
CS	chondroitin sulfate
ERT	enzyme replacement therapy
BMI	body mass index
*z* score	standard deviation score

## References

[1] Neufield E.F., Muenzer J., Scriver C., Beaudet A.L., Valle D., Sly W.S. (2001). The mucopolysaccharidoses. The Metabolic and Molecular Bases of Inherited Disease.

[2] Muenzer J. (2004). The mucopolysaccharidoses: A heterogeneous group of disorders with variable pediatric presentations. J. Pediatr..

[3] Muenzer J. (2011). Overview of the mucopolysaccharidoses. Rheumatology.

[4] Chuang C.K., Lin S.P., Sankar S. (2007). Neurochemical changes and therapeutical approaches in mucopolysaccharidoses. Neurochemistry of Metabolic Diseases-Lysosomal Storage Diseases, Phenylketouria and Canavan Disease.

[5] Tomatsu S., Montaño A.M., Oikawa H., Giugliani R., Harmatz P., Smith M., Suzuki Y., Orii T. (2012). Impairment of body growth in mucopolysaccharidoses. Handbook of Growth and Growth Monitoring in Health and Disease.

[6] Giugliani R., Federhen A., Vairo F., Vanzella C., Pasqualim G., da Silva L.M., Giugliani L., de Boer A.P., de Souza C.F., Matte U. (2016). Emerging drugs for the treatment of mucopolysaccharidoses. Expert. Opin. Emerg. Drugs.

[7] Różdżyńska-Świątkowska A., Jurecka A., Cieślik J., Tylki-Szymańska A. (2015). Growth patterns in children with mucopolysaccharidosis, I.; II. World J. Pediatr..

[8] Gardner C.J., Robinson N., Meadows T., Wynn R., Will A., Mercer J., Church H.J., Tylee K., Wraith J.E., Clayton P.E. (2011). Growth, final height and endocrine sequelae in a UK population of patients with Hurler syndrome (MPS1H). J. Inherit. Metab. Dis..

[9] D’Aco K., Underhill L., Rangachari L., Arn P., Cox G.F., Giugliani R., Okuyama T., Wijburg F., Kaplan P. (2012). Diagnosis and treatment trends in mucopolysaccharidosis I: Findings from the MPS I Registry. Eur. J. Pediatr..

[10] Rozdzynska A., Tylki-Szymanska A., Jurecka A., Cieslik J. (2011). Growth pattern growth prediction of body height in children with mucopolysaccharidosis type, I.I. Acta Paediatr..

[11] Patel P., Suzuki Y., Maeda M., Yasuda E., Shimada T., Orii K.E., Orii T., Tomatsu S. (2014). Growth charts for patients with Hunter syndrome. Mol. Genet. Metab. Rep..

[12] Parini R., Jones S.A., Harmatz P.R., Giugliani R., Mendelsohn N.J. (2016). The natural history of growth in patients with Hunter syndrome: Data from the Hunter Outcome Survey (HOS). Mol. Genet. Metab..

[13] Cho S.Y., Huh R., Chang M.S., Lee J., Kwun Y., Maeng S.H., Kim S.J., Sohn Y.B., Park S.W., Kwon E.K. (2014). Impact of enzyme replacement therapy on linear growth in Korean patients with mucopolysaccharidosis type II (Hunter syndrome). J. Korean Med. Sci..

[14] Żuber Z., Różdżyńska-Świątkowska A., Jurecka A., Tylki-Szymańska A. (2014). The effect of recombinant human iduronate-2-sulfatase (Idursulfase) on growth in young patients with mucopolysaccharidosis type II. PLoS ONE.

[15] Jones S.A., Parini R., Harmatz P., Giugliani R., Fang J., Mendelsohn N.J. (2013). HOS Natural History Working Group on behalf of HOS Investigators: The effect of idursulfase on growth in patients with Hunter syndrome: Data from the Hunter Outcome Survey (HOS). Mol. Genet. Metab..

[16] Muschol N.M., Pape D., Kossow K., Ullrich K., Arash-Kaps L., Hennermann J.B., Stücker R., Breyer S.R. (2019). Growth charts for patients with Sanfilippo syndrome (Mucopolysaccharidosis type III). Orphanet. J. Rare. Dis..

[17] de Ruijter J., Broere L., Mulder M.F., van der Ploeg A.T., Rubio-Gozalbo M.E., Wortmann S.B., Visser G., Wijburg F.A. (2014). Growth in patients with mucopolysaccharidosis type III (Sanfilippo disease). J. Inherit. Metab. Dis..

[18] Truxal K.V., Fu H., McCarty D.M., McNally K.A., Kunkler K.L., Zumberge N.A., Martin L., Aylward S.C., Alfano L.N., Berry K.M. (2016). A prospective one-year natural history study of mucopolysaccharidosis types IIIA and IIIB: Implications for clinical trial design. Mol. Genet. Metab..

[19] Melbouci M., Mason R.W., Suzuki Y., Fukao T., Orii T., Tomatsu S. (2018). Growth impairment in mucopolysaccharidoses. Mol. Genet. Metab..

[20] Montaño A.M., Tomatsu S., Brusius A., Smith M., Orii T. (2008). Growth charts for patients affected with Morquio A disease. Am. J. Med. Genet. A.

[21] Harmatz P., Mengel K.E., Giugliani R., Valayannopoulos V., Lin S.P., Parini R., Guffon N., Burton B.K., Hendriksz C.J., Mitchell J. (2013). The Morquio A Clinical Assessment Program: Baseline results illustrating progressive, multisystemic clinical impairments in Morquio A subjects. Mol. Genet. Metab..

[22] Quartel A., Hendriksz C.J., Parini R., Graham S., Lin P., Harmatz P. (2015). Growth Charts for Individuals with Mucopolysaccharidosis VI (Maroteaux-Lamy Syndrome). JIMD Rep..

[23] Chuang C.K., Lin S.P., Chung S.F. (2001). Diagnostic screening for mucopolysaccharidoses by the dimethylmethylene blue method and two dimensional electrophoresis. Zhonghua Yi Xue Za Zhi (Taipei).

[24] Chen W., Chang M.H. (2010). New growth charts for Taiwanese children and adolescents based on World Health Organization standards and health-related physical fitness. Pediatr. Neonatol..

[25] Lin H.Y., Lee C.L., Lo Y.T., Wang T.J., Huang S.F., Chen T.L., Wang Y.S., Niu D.M., Chuang C.K., Lin S.P. (2018). The Relationships Between Urinary Glycosaminoglycan Levels and Phenotypes of Mucopolysaccharidoses. Mol. Genet. Genomic Med..

[26] Tomatsu S., Shimada T., Mason R.W., Montaño A.M., Kelly J., LaMarr W.A., Kubaski F., Giugliani R., Guha A., Yasuda E. (2014). Establishment of glycosaminoglycan assays for mucopolysaccharidoses. Metabolites.

[27] Auray-Blais C., Lavoie P., Tomatsu S., Valayannopoulos V., Mitchell J.J., Raiman J., Beaudoin M., Maranda B., Clarke J.T. (2016). UPLC-MS/MS detection of disaccharides derived from glycosaminoglycans as biomarkers of mucopolysaccharidoses. Anal. Chim. Acta.

[28] Mashima R., Sakai E., Tanaka M., Kosuga M., Okuyama T. (2016). The levels of urinary glycosaminoglycans of patients with attenuated and severe type of mucopolysaccharidosis II determined by liquid chromatography-tandem mass spectrometry. Mol. Genet. Metab. Rep..

[29] Settembre C., Arteaga-Solis E., Ballabio A., Karsenty G. (2009). Self-eating in skeletal development: Implications for lysosomal storage disorders. Autophagy.

[30] Montaño A.M., Tomatsu S., Gottesman G.S., Smith M., Orii T. (2007). International Morquio A Registry: Clinical manifestation and natural course of Morquio A disease. J. Inherit. Metab. Dis..

